# Prediction of premature all-cause mortality in patients receiving peritoneal dialysis using modified artificial neural networks

**DOI:** 10.18632/aging.203033

**Published:** 2021-05-13

**Authors:** Qiongxiu Zhou, Xiaohan You, Haiyan Dong, Zhe Lin, Yanling Shi, Zhen Su, Rongrong Shao, Chaosheng Chen, Ji Zhang

**Affiliations:** 1 Department of Nephrology, The First Affiliated Hospital of Wenzhou Medical University, Wenzhou, Zhejiang, P.R. China; 2 Department of Nephrology, The First Affiliated Hospital of Soochow University, Jiangsu, P.R. China; 3 Department of Nephrology, Longgang Renmin Hospital, Wenzhou, Zhejiang, P.R. China

**Keywords:** all-cause mortality, peritoneal dialysis, artificial neural networks, age, risk factors

## Abstract

Premature all-cause mortality is high in patients receiving peritoneal dialysis (PD). The accurate and early prediction of mortality is critical and difficult. Three prediction models, the logistic regression (LR) model, artificial neural network (ANN) classic model and a new structured ANN model (ANN mixed model), were constructed and evaluated using a receiver operating characteristic (ROC) curve analysis. The permutation feature importance was used to interpret the important features in the ANN models. Eight hundred fifty-nine patients were enrolled in the study. The LR model performed slightly better than the other two ANN models on the test dataset; however, in the total dataset, the ANN models fit much better. The ANN mixed model showed the best prediction performance, with area under the ROC curves (AUROCs) of 0.8 and 0.79 for the 6-month and 12-month datasets. Our study showed that age, diastolic blood pressure (DBP), and low-density lipoprotein cholesterol (LDL-c) levels were common risk factors for premature mortality in patients receiving PD. Our ANN mixed model had incomparable advantages in fitting the overall data characteristics, and age is a steady risk factor for premature mortality in patients undergoing PD. Otherwise, DBP and LDL-c levels should receive more attention for all-cause mortality during follow-up.

## INTRODUCTION

The incidence and prevalence of end-stage renal disease (ESRD) has increased continually worldwide, and patients with ESRD are at higher risk of morbidity and mortality [1, 2]. Peritoneal dialysis (PD) is an established and cost-effective therapy for patients with ESRD [3, 4]. Although the mortality rates of patients receiving PD have decreased, their long-term survival remains poor [1, 5, 6]. Patients with a high risk of premature mortality who are undergoing PD should be managed with active treatment strategies to improve long-term survival. However, an early and accurate prediction of the risk of premature mortality in patients receiving PD is still difficult to achieve.

Barrett et al. attempted to predict early death in patients treated with dialysis using a scoring system based on a logistic regression (LR) model, but the authors found it impossible to accurately predict early death [7]. Although LR models are the most widely used methods for predicting binary medical outcomes, they are generalized linear models that require an assumption of a linear relationship between the transformed response in terms of the link function and the explanatory variables, which is not always suitable for medical datasets [8–10]. Artificial neural networks (ANNs), a type of machine learning algorithm, have become popular and helpful models for medical predictions, including nephrology [11]. ANNs automatically recognize complex nonlinear relationships and have become relatively competitive with conventional regression and statistical models in terms of usefulness [12]. However, the structure of an ANN requires an elaborate organization and adjustment to obtain the best performance.

Thus, the purpose of this study was to construct early prediction models based on the LR model and ANN model for all-cause premature mortality and compare the performance of the constructed models to select the most accurate models to predict the premature all-cause mortality in patients receiving PD.

## MATERIALS AND METHODS

### Study population

Data from 1241 patients with ESRD who initially started PD between Jan 2006 and Dec 2019 at the First Affiliated Hospital of Wenzhou University were collected and reviewed. The inclusion criteria were as follows: 1. older than 18 years and 2. routine follow-up for more than twelve months in our PD center. The exclusion criteria were as follows: 1. a history of continuous hemodialysis for more than six months before continuous ambulatory peritoneal dialysis (CAPD) or a combination of continuous hemodialysis and CAPD, 2. a history of kidney transplantation, and 3. missing important data. Patients who met the above criteria were eventually enrolled in this study. The study protocol was reviewed and approved by the Ethics Committee of the First Affiliated Hospital of Wenzhou University before collecting any data.

### Data collection and preparation

The following clinical characteristics were collected at the initiation of CAPD as predictor variables: demographic variables, including sex, age and complications such as chronic heart disease (CHD), diabetes mellitus (DM), and malignancy; and laboratory variables, including systolic blood pressure (SBP, mmHg), diastolic blood pressure (DBP, mmHg), total triglycerides (Tg), total cholesterol (Tc), low-density lipoprotein cholesterol (LDL-c), high-density lipoprotein cholesterol (HDL-c), serum albumin (g/dL), hemoglobin (g/dL), blood urea nitrogen (BUN, mg/dL), serum creatinine (Scr, μmol/L), serum phosphorus (P, mmol/l), intact parathyroid hormone (iPTH, pg/ml), and Kt/V. The causes of premature death were recorded during follow-up, and the primary endpoint of the study was all-cause mortality. We collected the data at the beginning of PD and during the follow-up period. Three datasets, namely, the 0-month, 6-month, and 12-month datasets, were collected, and the 0-month dataset (also called the total dataset) was used for training the prediction models. Missing values were imputed with values from the nearest three months. All included numerical variables were normalized by the Z-score.

### Construction of prediction models

The TensorFlow platform (https://www.tensorflow.org/) was used for training the ANN models [13]. We constructed two different types of ANN models. One is called the ANN classic model, which was built using a single neural network with 12 hidden layers. The numerical variables and categorical variables were input into the neural network simultaneously (Supplementary Figure 1). The other is called the ANN mixed model. Two different sub-neural networks were built for the numerical variables and categorical variables with nine hidden layers and eleven hidden layers, respectively. The two sub-networks were then merged into a new neural network with two hidden layers for predicting the outcomes (Supplementary Figure 1). The hyperparameters of the ANN models were adjusted during the study. Finally, we set the following parameters for the ANN models: epoch = 3500, batch size = 220, iteration = 0.0001, and L1 and L2 regularization penalties. The multivariable logistic model was built using the Scikit-learn platform [14].

We selected the 0-month dataset to train the ANN models and logistic models and construct an early prediction model. The full 0-month dataset was randomly divided into three datasets: a training dataset (63.2%), validation dataset (48%), and test dataset (20%). The training dataset was used to train the ANN models and logistic models. The validation dataset displayed 31.2% overlap with the training dataset and was used to control overfitting during training of the ANN model. The test dataset did not have any overlapping data with the training dataset, and the validation dataset was used to assess the performance of the ANN models and logistic models (Supplementary Figure 2).

### Evaluation of the performance of the ANN and logistic models

We calculated the predictive outcomes of the ANN and logistic models using the test dataset and the 0-month, 6-month, and 12-month datasets during the construction of every model. Then, the areas under the receiver operating characteristic (ROC) curves (AUROCs) were calculated to filter models with extremely poor performance using a threshold of 0.6, and ROC curves were plotted to visualize the relationship between the true positive rate (TPR) and false positive rate (FPR) at different cutoff values. We also calculated the accuracy, F1 score, precision, and recall values at a fixed threshold value (0.2) to evaluate the performance of the selected models in predicting positive cases (dead patients) or negative cases (surviving patients) using the Scikit-learn application [15]. A phi coefficient analysis was performed to measure the association between the predicted and true outcomes [16]. The permutation feature importance, which is defined as the decrease in the score of a model when a single feature value is randomly shuffled [17], was calculated to evaluate the significance of the included variables.

### Statistical analysis

The numerical data are presented as the means [standard deviations (SD)] or the medians [interquartile ranges (IQRs)], and differences between the groups were examined using variance analysis or the Kruskal-Wallis rank test. Categorical data are presented as counts with percentages (%), and differences between the groups were analyzed using Pearson’s chi-square test. Multivariable LR models based on the 0-month, 6-month, and 12-month datasets were built to evaluate the effects of the included variables on the primary outcomes. All reported p-values are two-tailed, and p-values less than 0.05 were considered to indicate a statistically significant difference. Python (version 3.8) [18] and R software (version 4.0.2, R Core Team) [19] and embedded packages were used to prepare the datasets, perform the analyses, and create the plots [20–24]. P<0.05 was set as statistically significant.

## RESULTS

Eight hundred fifty-nine patients who met the criteria were enrolled in the study, and 82 (9.54%) patients met the primary endpoint at a median follow-up time of 40.5 [18.2, 59.8] months. According to our 0-month dataset, the variables diabetes, CHD, age, DBP, LDL-c levels, and serum albumin levels were significantly different between the patients with and without the primary endpoint (Table 1). Supplementary Figure 3 displays the comparisons of the included variables in the 0-month, 6-month, and 12-month datasets. The plots of the three datasets showed similar differences in most of the included variables.

**Table 1:** Baseline characteristics of the included patients with CAPD.

**Characteristics**	**All-cause premature mortality**		**p-value**
	**No**	**Yes**	
Case (n)	777	82	
Age (years, median [IQR])	48.0 [38.0, 58.0]	63.0 [54.0, 70.0]	<0.001
Male (n, %)	432 (55.6)	51 (62.2)	0.3
SBP (mmHg, median [IQR])	145.0 [132.0, 159.0]	148.0 [133.2, 164.0]	0.2
DBP (mmHg, median [IQR])	88.0 [78.0, 97.0]	79.0 [71.0, 91.5]	<0.001
Tg (mmol/L, median [IQR])	1.6 [1.2, 2.1]	1.6 [1.2, 2.1]	0.9
Tc (mmol/L, median [IQR])	4.6 [3.9, 5.4]	4.8 [4.1, 5.5]	0.3
LDL-c (mmol/L, median [IQR])	2.5 [2.0, 3.1]	2.8 [2.2, 3.4]	0.01
HDL-c (mmol/L, median [IQR])	1.0 [0.9, 1.2]	1.0 [0.8, 1.2]	0.05
Serum albumin (g/L, median [IQR])	37.1 [33.5, 40.4]	35.1 [32.0, 39.0]	0.004
Hemoglobin (g/L, median [IQR])	95.0 [82.0, 108.0]	93.0 [81.2, 104.0]	0.2
BUN (mmol/L, median [IQR])	19.1 [13.9, 24.6]	18.8 [12.9, 24.8]	0.9
SCR (μmol/L, median [IQR])	646.0 [326.0, 888.0]	526.5 [306.2, 773.5]	0.05
Serum calcium (mmol/L, median [IQR])	2.2 [2.0, 2.3]	2.1 [2.0, 2.2]	0.3
Serum phosphorus (mmol/L, median [IQR])	1.6 [1.3, 1.8]	1.5 [1.3, 1.8]	0.3
iPTH (pg/mL, median [IQR])	212.6 [108.4, 371.9]	181.6 [82.0, 309.4]	0.07
Kt/V (median [IQR])	1.9 [1.7, 2.2]	1.8 [1.6, 2.1]	0.1
Diabetes (n, %)	176 (22.7)	37 (45.1)	<0.001
Hypertension (n, %)	619 (94.8)	78 (98.7)	0.2
Chronic heart disease (n, %)	178 (22.9)	38 (46.3)	<0.001
Malignancy (n, %)	53 (6.8)	10 (12.2)	0.1
Follow-up time (month, median [IQR])	38.0 [16.0, 66.0]	40.5 [18.2, 59.8]	0.9

SBP: systolic blood pressure; DBP: diastolic blood pressure; Tg: triglycerides; Tc: total cholesterol; LDL-c: low-density lipoprotein cholesterol; HDL-c: high-density lipoprotein cholesterol; BUN: urea nitrogen; SCR: serum creatinine; iPTH: intact parathyroid hormone.

Our three multivariable logistic models based on the three full datasets named model 0, model 1, and model 2 showed that age (model 0: β (se) = 0.071 (0.013), p-value < 0.001; model 1: β (se) = 0.065 (0.013), p-value < 0.001; model 2: β (se) = 0.068 (0.013), p-value < 0.001) was a steadily significant risk factor for the primary outcome. In addition, the complication of CHD (model 0: β (se) =0.578 (0.272), p-value = 0.03; model 1: β (se) = 0.479 (0.276), p-value = 0.08; model 2: β (se) = 0.462 (0.274), p-value = 0.09), serum albumin levels (model 0: β (se) = -0.041 (0.029), p-value = 0.2; model 1: β (se) = -0.1 (0.033), p-value = 0.003; model 2: β (se) = -0.117 (0.034), p-value = 0.001), and LDL-c levels (model 0: β (se) = 0.514 (0.309), p-value = 0.1; model 1: β (se) = 0.87 (0.412), p-value = 0.04; model 2: β (se) = 0.791 (0.36), p-value = 0.03) were also significantly associated with the primary outcome in the different datasets (Table 2).

**Table 2:** Multivariable logistic regression models for the three full datasets.

**Variables**	**Model 0**		**Model 1**		**Model 2**	
	**β (se)**	**p-value**	**β (se)**	**p-value**	**β (se)**	**p-value**
Age	0.071 (0.013)	<0.001*	0.065 (0.013)	<0.001*	0.068 (0.013)	<0.001*
CHD	0.578 (0.272)	0.03*	0.479 (0.276)	0.08	0.462 (0.274)	0.09
DBP	-0.014 (0.012)	0.2	-0.028 (0.015)	0.07	-0.026 (0.016)	0.1
Diabetes	0.355 (0.294)	0.2	0.051 (0.305)	0.9	-0.023 (0.315)	0.9
Malignancy	0.342 (0.416)	0.4	0.272 (0.416)	0.5	0.308 (0.411)	0.5
Albumin	-0.041 (0.029)	0.2	-0.1 (0.033)	0.003*	-0.117 (0.034)	0.001*
BUN	0 (0.024)	1	0.006 (0.028)	0.8	-0.015 (0.031)	0.6
Ca	0.854 (0.682)	0.2	1.469 (0.876)	0.09	1.379 (0.87)	0.1
SCR	-0.001 (0.001)	0.2	-0.001 (0.001)	0.2	0 (0.001)	0.4
Hb	-0.014 (0.008)	0.09	-0.015 (0.009)	0.09	-0.013 (0.009)	0.2
HDL-c	-0.457 (0.562)	0.4	-0.162 (0.632)	0.8	0.345 (0.264)	0.2
LDL-c	0.514 (0.309)	0.1	0.87 (0.412)	0.04*	0.791 (0.36)	0.03*
P	0.478 (0.423)	0.3	0.343 (0.49)	0.5	0.488 (0.51)	0.3
iPTH	0.054 (0.158)	0.7	0.061 (0.172)	0.7	0.12 (0.178)	0.5
Tc	-0.134 (0.272)	0.6	-0.532 (0.364)	0.1	-0.554 (0.289)	0.06
Tg	-0.177 (0.192)	0.4	0.2 (0.216)	0.4	0.282 (0.173)	0.1
SBP	0.012 (0.007)	0.1	0.014 (0.009)	0.1	0.015 (0.009)	0.1
Sex	0.293 (0.291)	0.3	0.421 (0.318)	0.2	0.56 (0.321)	0.08
Kt/V	-0.259 (0.313)	0.4	0 (0.376)	1	0.124 (0.395)	0.8

Model 0: 0-month datasets; Model 1: 6-month datasets; Model 2: 12-month datasets; iPTH: intact parathyroid hormone; SBP: systolic blood pressure; DBP: diastolic blood pressure; MAP: mean arterial pressure; BMI: body mass index; RAAS: renin–angiotensin–aldosterone system agents; CCBs: calcium channel blockers.

**Figure 1:**
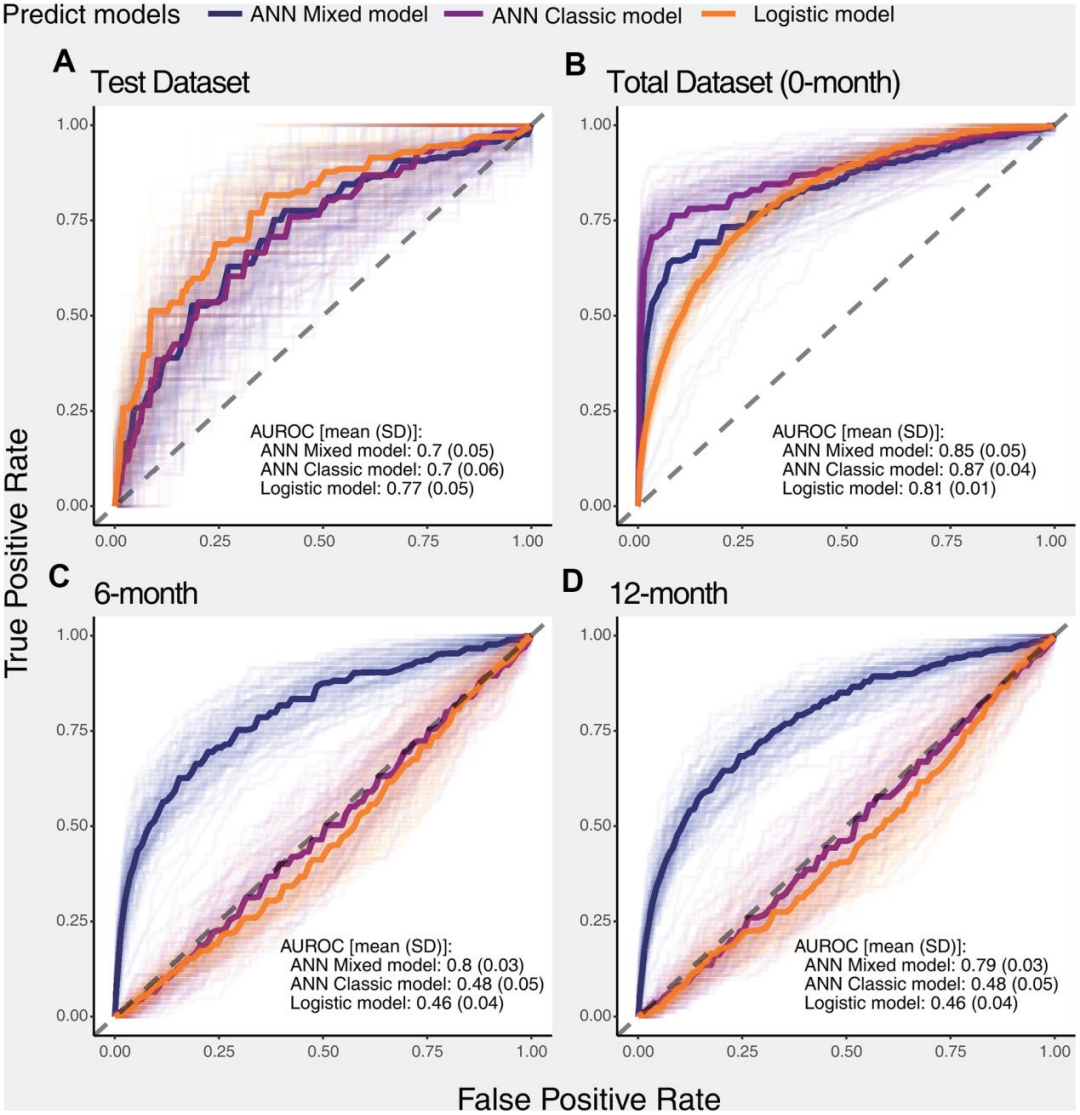
**ROC curves of selected models for predicting the primary outcome in different datasets.** The dark solid lines indicate the median curve of the three types of models (ANN mixed model, ANN classic model, and logistic model). (**A**) Performance of selected models in the test dataset, (**B**) Performance of selected models in the total dataset, (**C**) Performance of selected models in the 6-month dataset, (**D**) Performance of selected models in the 12-month dataset.

We constructed 100 ANN classic models, 100 ANN mixed models, and 100 logistic models using the 0-month dataset. The accuracy and loss function values per epoch are displayed in Supplementary Figure 4, and slight overfitting was observed in both the ANN classic model and ANN mixed model. ANN models with poor performance (i.e., AUROC values of less than 0.6) were filtered. According to the ROC curves, the logistic model showed better performance than the ANN models on the test dataset, but the ANN models fit more perfectly in the total dataset. Importantly, in the 6-month and 12-month datasets, the ANN mixed model showed excellent performance compared with both the ANN classic model and logistic model, while the ANN classic model and logistic model showed similar performance outcomes (Figure 1).

**Figure 2:**
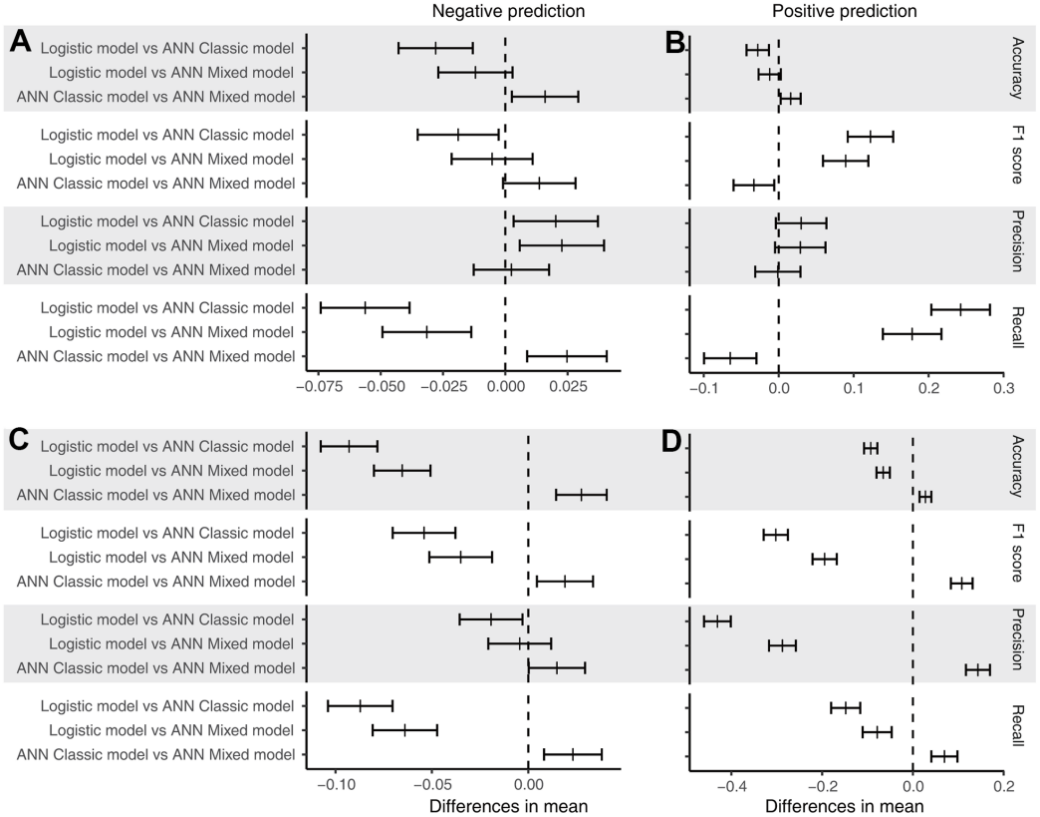
**Post hoc test of performance.** (**A**) Performance of the models for the negative prediction in the test dataset; (**B**) performance of the models for the positive prediction in the test dataset; (**C**) performance of the models for the negative prediction in the total dataset; and (**D**) performance of the models for the positive prediction in the total dataset. The short bar indicates the difference in the mean value with a 95% confidence interval.

**Figure 3:**
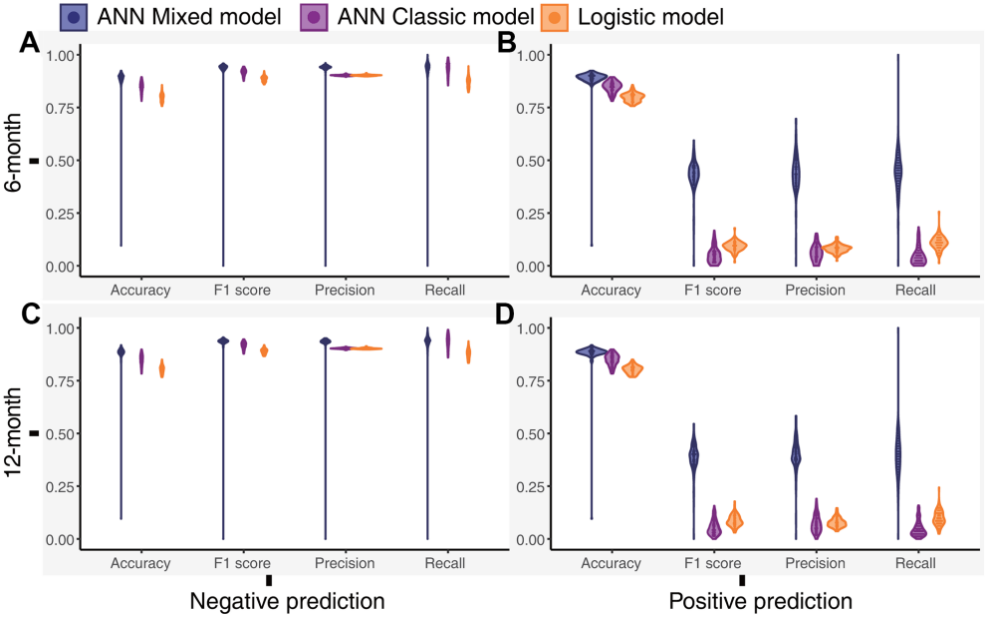
**Distribution of the performance outcomes of the models for the 6-month and 12-month datasets.** (**A**) Performance of the models for the negative prediction in the 6-month dataset, (**B**) Performance of the models for the positive prediction in the 6-month dataset, (**C**) Performance of the models for the negative prediction in the 12-month dataset, (**D**) Performance of the models for the positive prediction in the 12-month dataset.

We calculated the accuracy, F1 score, precision, and recall of positive and negative predictions using a fixed threshold of 0.2 for the test datasets, total dataset (0-month dataset), 6-month dataset, and 12-month dataset. The logistic model showed superior positive prediction in the test datasets compared with the ANN models (Figure 2A, 2B). However, the performance of the ANN models was significantly better than that of the logistic models when analyzing the total dataset (Figure 2C, 2D). Furthermore, the ANN classic models fit the total dataset better than the ANN mixed models. The ANN mixed models performed excellently in predicting premature all-cause mortality compared with the logistic model and ANN classic model in our follow-up datasets (Figure 3). Notably, the positive prediction may be more important for clinical practice. The mean precision and recall for the positive prediction of the ANN mixed models were 0.44 (0.09) and 0.44 (0.1) in the 6-month dataset and 0.4 (0.07) and 0.39 (0.10) in the 12-month dataset, respectively, but these values were significantly higher than those of the ANN classic models and logistic models (Table 3), consistent with the results of the ROC analysis.

**Table 3:** Performance of the models in the follow-up datasets.

**Performance**	**Negative prediction**			**p-value**	**Positive prediction**			**p-value**
	**ANN mixed model**	**ANN classic model**	**Logistic model**		**ANN mixed model**	**ANN classic model**	**Logistic model**	
**6-month**								
Accuracy	0.89 (0.07)	0.85 (0.03)	0.80 (0.02)	<0.001	0.89 (0.07)	0.85 (0.03)	0.80 (0.02)	<0.001
F1 score	0.93 (0.08)	0.92 (0.02)	0.89 (0.01)	<0.001	0.43 (0.07)	0.05 (0.04)	0.09 (0.02)	<0.001
Precision	0.93 (0.08)	0.90 (0.00)	0.90 (0.00)	<0.001	0.44 (0.09)	0.06 (0.04)	0.08 (0.02)	<0.001
Recall	0.93 (0.08)	0.93 (0.03)	0.87 (0.02)	<0.001	0.44 (0.10)	0.05 (0.04)	0.11 (0.03)	<0.001
**12-month**								
Accuracy	0.88 (0.07)	0.85 (0.03)	0.80 (0.02)	<0.001	0.88 (0.07)	0.85 (0.03)	0.80 (0.02)	<0.001
F1 score	0.93 (0.08)	0.92 (0.02)	0.89 (0.01)	<0.001	0.39 (0.07)	0.05 (0.03)	0.09 (0.03)	<0.001
Precision	0.93 (0.08)	0.90 (0.00)	0.90 (0.00)	<0.001	0.40 (0.07)	0.07 (0.04)	0.08 (0.02)	<0.001
Recall	0.93 (0.08)	0.93 (0.03)	0.88 (0.02)	<0.001	0.39 (0.10)	0.05 (0.04)	0.10 (0.04)	<0.001

Values are presented as the means (SDs).

**Figure 4:**
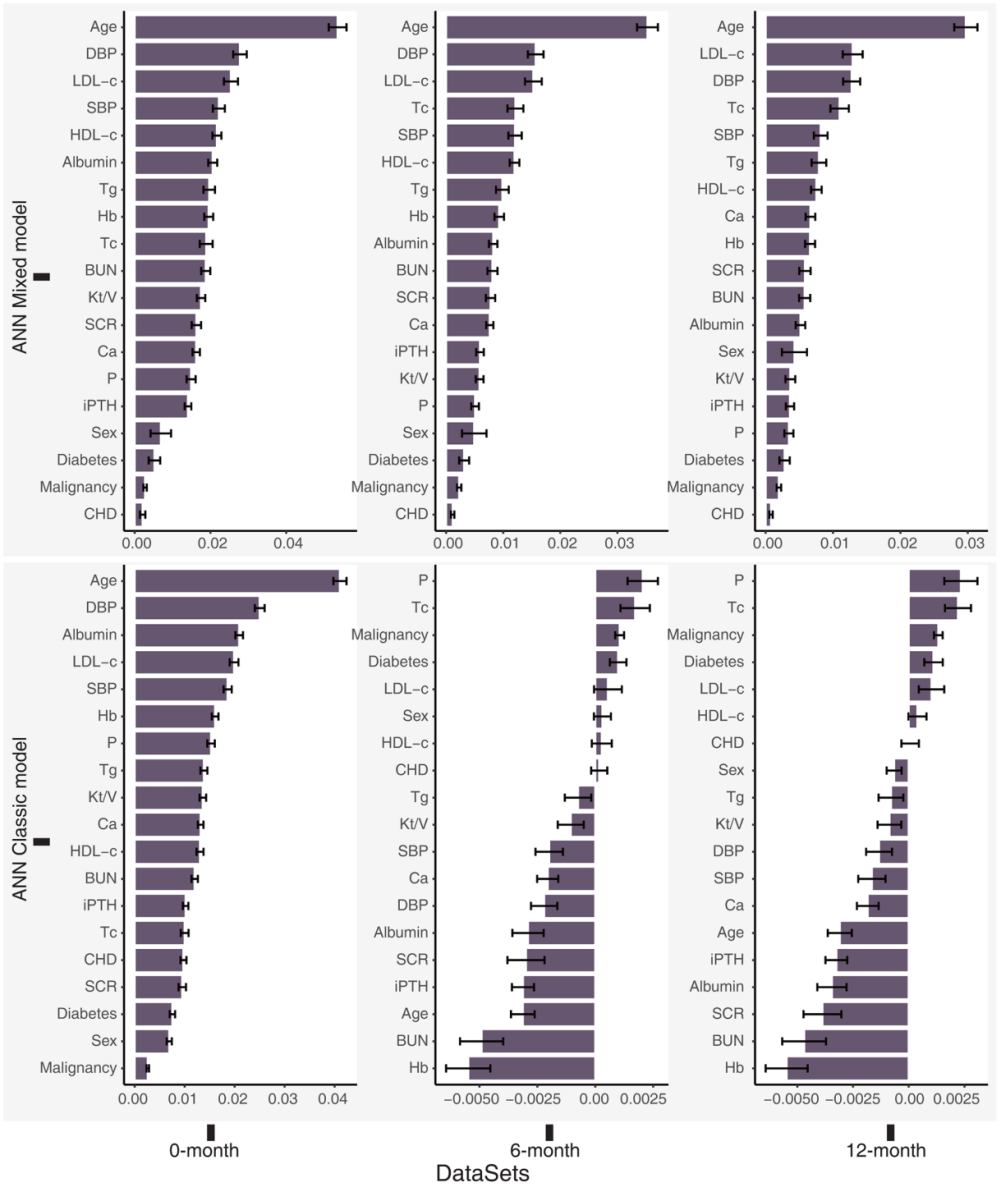
**Permutation feature importance for the ANN models in the total dataset (0-month), 6-month dataset and 12-month dataset.** Higher positive values indicated greater importance of the model, and negative values may indicate that the feature is worse than noise.

We identified age as an essential stable variable predicting death in patients treated with PD. The first three critical features in our ANN mixed model were age, DBP, and LDL-c levels. Furthermore, the features may exert negative effects (worse than noise) on the ANN classic models, indicating that the ANN classic model performed poorly on the 6-month and 12-month datasets (Figure 4).

## DISCUSSION

According to our baseline dataset, the traditional risk factors age, diabetes, albumin level and cardiovascular disease were significantly different between the surviving patients and patients who experienced premature mortality, consistent with the findings of previous studies [25, 26]. Furthermore, DBP and LDL-c levels were also significantly different between the two groups. Our multivariable LR models based on the baseline, 6-month and 12-month datasets further confirmed that an older age combined with cardiovascular disease, lower serum albumin levels, and higher LDL-c levels were independent risk factors for premature mortality in patients receiving PD.

Different performance outcomes of the LR models and ANN models for the test dataset and whole dataset were observed in our study. This difference may be attributed to the different algorithms used by LR and ANN. LR is a linear classification method, and its cost function is convex. Thus, it is guaranteed to find the global cost minimum [27, 28]. Although the ANN model is a nonlinear classification model and can fit perfectly to the training dataset, the cost function of a neural network is generally neither convex nor concave, and it easily falls into a local optimum [29]. Thus, the ANN model displayed an inferior performance compared to the LR model when analyzing a small sample but fit better in a large-scale population.

Papadrakakis et al. found that the performance of the ANN model can be significantly improved by adjusting the network structure and hyperparameters of the model [30]. Our study developed a new structure for the ANN model, which was called the ANN mixed model. Our external validation of the follow-up dataset showed the predictive performance increased significantly using the ANN mixed model to analyze the 6-month and 12-month datasets than using the LR model and ANN classic model. Thus, we considered that the ANN mixed model has a higher efficiency of generalization performance. However, the mean precision and recall for the positive prediction of the ANN mixed models in the 6-month and 12-month datasets was approximately 40%, suggesting that our model might be insufficient to detect positive cases in an external dataset. One reason is the imbalanced category of premature all-cause mortality in our cohort, which significantly increased the difficulty of identifying the positive cases. Furthermore, the validation of the 6-month and 12-month datasets included patients who had been receiving treatment, and the treatment significantly affects the clinical characteristics of patients receiving PD, which potentially affected the prediction accuracy of our model.

The classic studies constructing prediction models or identifying risk factors mostly included categorical and continuous variables simultaneously [25, 31–33]. Our study showed that the ANN classic and LR models, which were similar to the classic studies, were inaccurate in the 6-month and 12-month datasets. Burrett et al. considered that differences in the populations studied may have contributed to the loss of predictive power for the prognostic score [7]. We assumed that the significance of the scalar was different between categorical variables and continuous variables. The simultaneous inclusion of categorical and continuous variables in an identical vector space for fitting a model may increase overfitting and adversely affect the generalization performance. Based on our results, the construction of separate vector spaces for categorical and continuous variables in a model significantly improved the generalization performance.

An ANN is a black-box model, and it does not easily display the relationship between features and outcomes [34]. We used a permutation feature importance analysis, which is used for interpreting the importance of variables in a model [35–37], to identify the important characteristics contributing to premature death. Importantly, age, DBP, and LDL-c levels were the top three important variables in the ANN mixed model. The LR models based on the 6-month and 12-month datasets also showed that DBP and LDL-c levels were independent risk factors for premature all-cause death. Sakacı et al. also found that age is an independent risk factor for mortality in patients undergoing dialysis [38]. Although age is an unmodifiable variable, some age-related variables, such as nutritional status, can still be improved by better management [39]. Previous studies have mainly focused on the significance of SBP in patients with ESRD [40]. Our research identified DBP as a crucial risk factor for predicting death in patients undergoing PD and one of the most valuable variables in the ANN model. Lip et al. observed a reverse J-shaped relationship between DBP and death from cardiovascular events. Cardiovascular death was also the primary factor contributing to premature mortality in our patients receiving PD [31, 41]. Therefore, DBP should receive more attention in patients receiving PD during clinical practice. Lowering LDL-c levels can significantly improve the prognosis of patients with chronic kidney disease (CKD) stage 1-4, but researchers have not clearly determined whether it can improve the prognosis of patients with CKD5 or CKD5d [42–44]. Strict lipid control may also cause malnutrition in patients receiving dialysis, which is an important factor contributing to the death of patients receiving dialysis [45, 46]. LDL-c levels were closely related to the premature mortality of patients treated with PD in our study. As the life span of patients treated with dialysis increases, the effect of dyslipidemia on patients receiving PD cannot be ignored. Therefore, further studies of the role of lipids in patients undergoing PD are still necessary.

Our research had some limitations. First, the study is based on a single center and a relatively insufficient sample size, which may contribute to overfitting and affect generalization performance. Although L1 and L2 regularization were used during ANN training and follow-up datasets were used for external validation, the initial PD data must still be collected from other centers for external verification. Second, a few patients receiving PD withdrew during follow-up, and these patients may have died at home or in other departments but were not categorized into the premature mortality group, resulting in an endpoint determination bias affecting the accuracy of our model. Third, the proportion of patients with premature all-cause mortality is small in our cohort, leading to a significant imbalance in classification, which affects the detection power of our model.

In summary, our study compared the value of traditional logistic models and ANNs in predicting all-cause mortality in patients treated with PD and showed that ANNs had incomparable advantages in fitting the overall data characteristics. Thus, a highly precise ANN model for the early prediction of premature all-cause mortality in patients receiving PD was established. Our study also showed the importance of DBP and LDL-c levels in predicting the premature all-cause mortality of patients receiving PD; thus, these factors should receive more attention during follow-up.

## Supplementary Material

Supplementary Figures
