# Supplementary material for: Prediction of premature all-cause mortality in patients receiving peritoneal dialysis using modified artificial neural networks
Source: Aging (Albany NY). 2021 May 13;13(10):14170–84. doi: 10.18632/aging.203033 (PMC8202888; doi:10.18632/aging.203033)
Supplement: Supplementary Figures [file aging-13-203033-s001.pdf]

SUPPLEMENTARY FIGURES

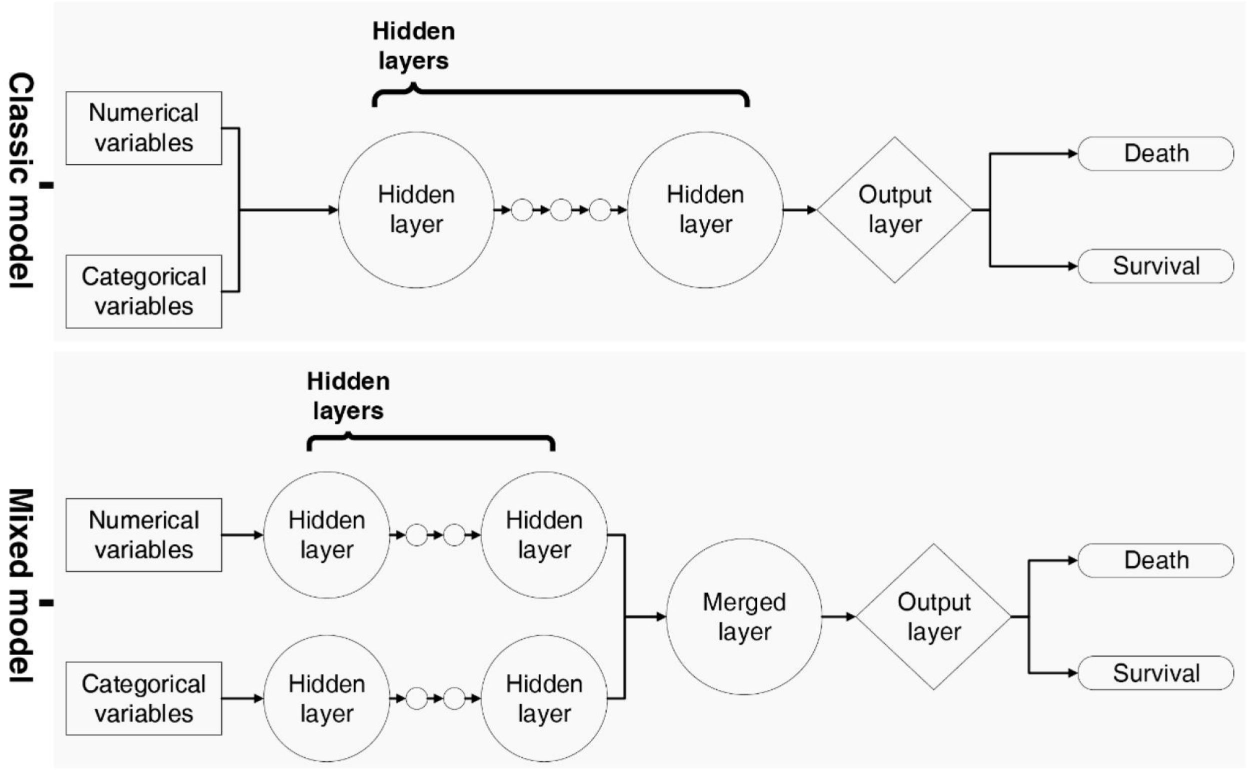

Supplementary Figure 1. Schematic diagram of the two types of neural networks.

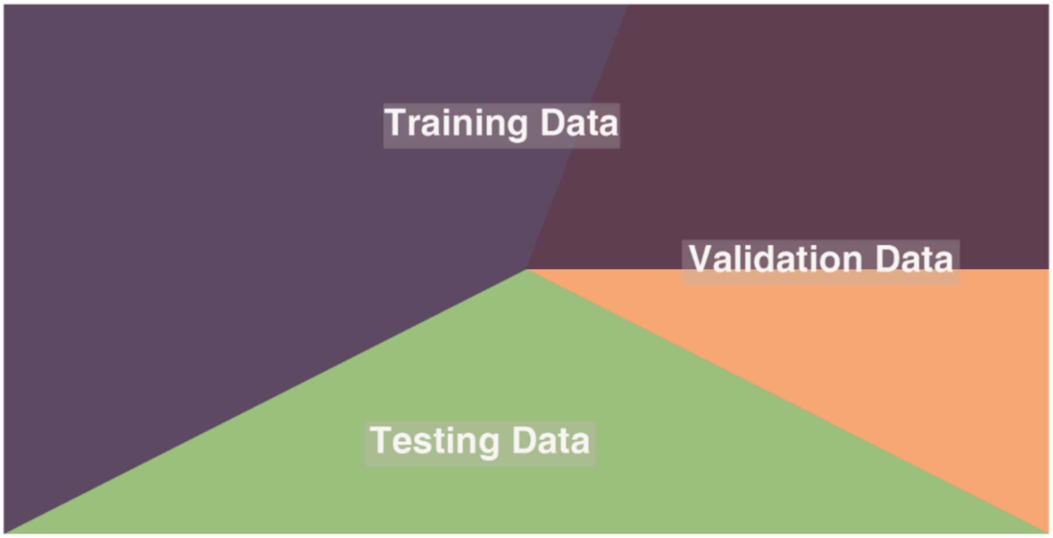

Supplementary Figure 2. Diagram of the dataset segmentation process during model training. The 0-month dataset (also called the total dataset) was used for training the ANN and logistic models and separated into three datasets for model training. Training dataset: blue plus purple; validation dataset: purple plus orange; test dataset: green.

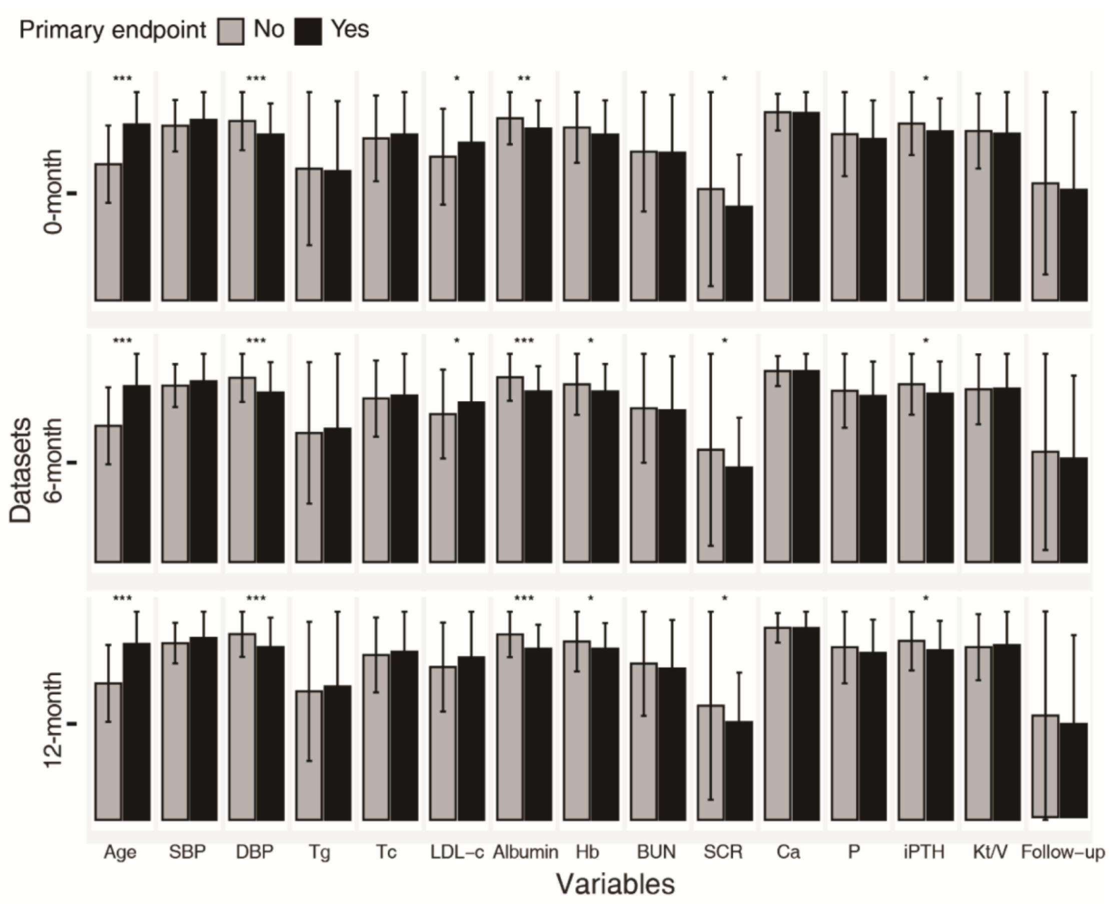

Supplementary Figure 3. The different mean values of numerical variables between surviving and non-surviving patients. The short bar indicates the SD. *, **, and *** indicate p-values less than 0.05, 0.01, and 0.001, respectively.

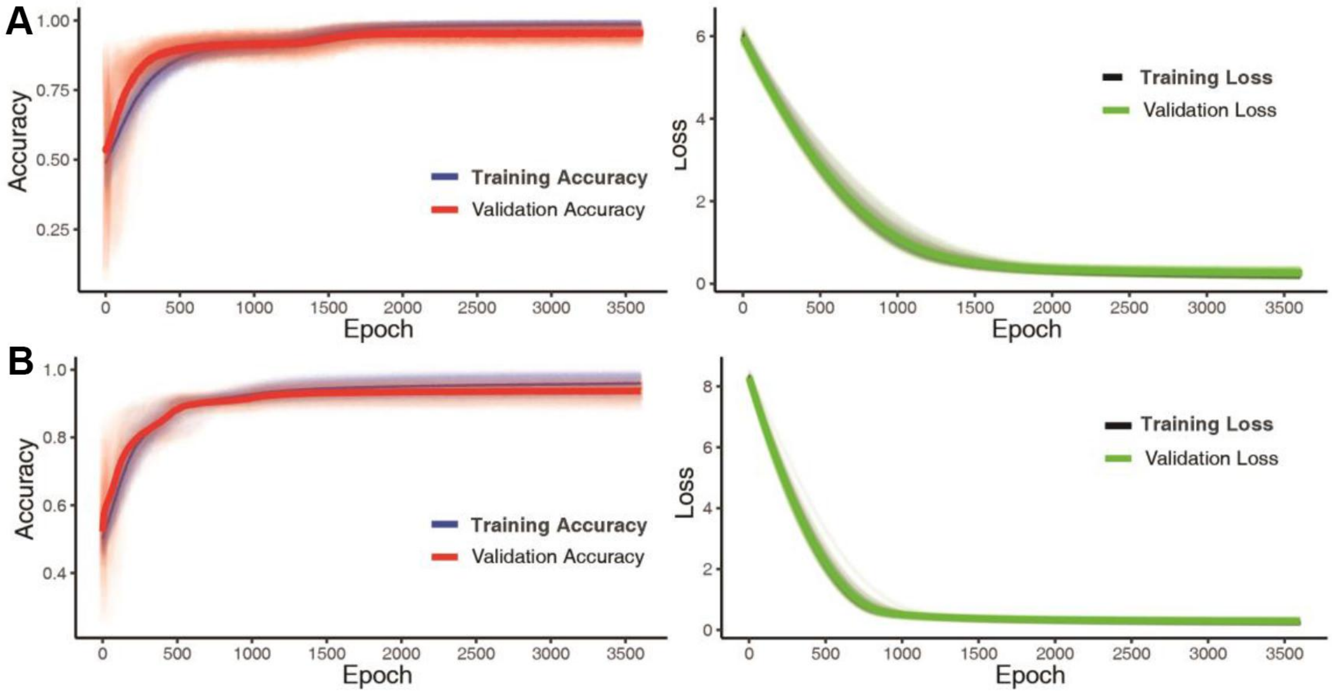

Supplementary Figure 4. Changes in the accuracy and loss function values during the training process. (**A**) ANN classic models; (**B**) ANN mixed models.
